# P-1676. Retrospective Evaluation of the Impact of Infectious Diseases Consultation on Prescribing Patterns and Clinical Outcomes among Patients with Gram-negative Bacteremia in the Community Hospital Setting

**DOI:** 10.1093/ofid/ofae631.1842

**Published:** 2025-01-29

**Authors:** Rowan Warehall, David Gothard, Amy Fabian

**Affiliations:** UH St John, Cleveland, Ohio; The MetroHealth System, Cleveland, Ohio; University Hospitals St. John Medical Center, Westlake, Ohio

## Abstract

**Background:**

Gram-negative bloodstream infections (BSI) are concerning due to high mortality rates reporting up to 30%; however, infectious diseases (ID) consultation has been associated with reduced mortality. According to consensus statement recommendations, fluoroquinolones (FQ) or trimethoprim-sulfamethoxazole (TMP/SMZ) are first-line oral treatment options and high bioavailable beta-lactams (HBBL) are alternatives for the treatment of Gram-negative BSI. The objective of this study is to evaluate differences in oral antibiotic prescribing patterns and outcomes between ID and non-ID providers among patients with Enterobacterales BSI in a community hospital setting.

**Methods:**

This is a multi-centered, retrospective cohort. Adult patients who received treatment for confirmed Enterobacterales BSI between January 1, 2022 and June 30, 2023 were included. Patients who completed treatment with only IV antibiotics, were greater than 89 years of age, elected palliative or hospice care before completion of treatment, died prior to receiving oral antibiotics, or with polymicrobial blood cultures were excluded. The primary outcome was the proportion of patients ordered FQ, TMP/SMZ, HBBL, or low-moderate bioavailable beta-lactam (LBBL) as oral stepdown. Secondary outcomes included length of stay, IV, oral, and total length of therapy (LOT), 30-day readmission, *Clostridioides difficile* infection (CDI), and all-cause mortality. The primary outcome was analyzed using a chi-square test with post hoc Bonferroni adjusted z tests to determine specific categorical differences.

**Results:**

In the ID consult group (n=95) 72.6% of patients received a FQ, 6.3% TMP/SMZ, 16.8% HBBL, and 4.2% LBBL whereas in the non-ID consult group (n=77) 50.6% received a FQ, 5.2% TMP/SMZ, 39% HBBL, and 5.2% LBBL (p=0.009; Bonferroni z test p< 0.0125 for FQ and HBBL). Secondary outcomes did not differ significantly between the two groups.

**Conclusion:**

FQ were more likely to be prescribed for oral stepdown in the ID consult group whereas HBBL were more likely to be prescribed in the non-ID consult group. Overall, among either group rates of prescribing were low for both TMP/SMZ and LBBL.

**Disclosures:**

**All Authors**: No reported disclosures

